# Peripheral body temperature impairment in individuals with type 1 diabetes mellitus

**DOI:** 10.5935/1984-0063.20180026

**Published:** 2018

**Authors:** Mark Thomaz Ugliara Barone, Bruno Gonçalves, Luiz Menna-Barreto

**Affiliations:** 1 Escola de Artes, Ciências e Humanidades da Universidade de São Paulo, Grupo Multidisciplinar de Desenvolvimento e Ritmos Biológicos (GMDRB) - São Paulo - SP - Brazil.; 2 ADJ Diabetes Brasil, Research and Education - São Paulo - SP - Brazil.

**Keywords:** Type 1 Diabetes Mellitus, Body Temperature, Diabetes Complications, Circadian Rhythm

## Abstract

**Objective:**

The aim of the present study was to evaluate the peripheral temperature rhythmicity and control in individuals with type 1 diabetes mellitus.

**Methods:**

Twelve non-obese adults (20-40 years old) with type 1 diabetes mellitus (T1D) and eight control individuals, matched for age and BMI, wore a wrist temperature recorder for 10 consecutive days. Recorded data were aggregated to calculate M10 (ten hours of highest temperature) and L5 (five hours of lowest temperature) of wrist temperature values for both groups.

**Results:**

Mean wrist temperature and M10 were not different when comparing the groups. The wrist temperature amplitude was reduced in the T1D group (*p*=0.039), due to a higher L5 (*p*=0.038).

**Discussion:**

While the higher L5 observed in T1D could be explained by less efficient heat dissipation, the amplitude flattening coincides with that observed in elderly.

## INTRODUCTION

Type 1 diabetes mellitus (T1D) is a complex chronic condition attributed to pancreatic beta-cells destruction, in most cases due to autoimmune reactions, which leads to insulin deficiency and the permanent need of exogenous insulin administration^1^. Abnormalities in temperature regulation and rhythm have been reported in human beings and animals with diabetes^2^^,^^3^. Body temperature in humans has been extensively studied due to the fact that it presents one of the most robust circadian rhythms^4^. Recently, different non-invasive routes were proposed, tested and some recommended with the objective of accessing this rhythm, such as the one used in the present study, wrist temperature^5^^,^^6^. Alterations in basic characteristics of the temperature or other rhythm’s amplitude, stability, synchronicity and/or period and phase length are commonly associated with psychological, neurological or metabolic impairments and/or aging^4^^,^^7^^-^^10^. Thermoregulation is known to be impaired in individuals with T1D, especially when associated with long disease duration, poor glycemic control, low aerobic fitness and neuropathy^3^. Most of these alterations are attributed to the reduced skin blood flow, cutaneous dilatation, sweating and thermosensitivity. The impact of this impairment on the temperature rhythm, a key signal for other rhythms^11^^,^^12^, was rarely studied^2^. For this reason we aimed to evaluate the peripheral temperature rhythm, with emphasis on it amplitude, in T1D and control individuals.

## MATERIALS AND METHODS

### Subjects

A subgroup of twelve out of the eighteen adults with T1D who participated in a previous study^13^ had their wrist temperature recorded and analyzed. They were all free of diabetes chronic complications and of any drugs that could affect sleep; all were non-obese (BMI > 18 and < 30 kg/m^2^) and aged between 20 and 40 years. They were not night- or shift-workers and had no previous diagnosis of sleep disorders. Chronic complications assessment included: retinal inspection by ophthalmologists, measurement of 24 hours microalbuminuria and creatinine levels, 10 g monofilament sensation, vibration perception with a 128-Hz tuning fork, resting heart rate, and blood pressure adaptation when standing up. In addition to the T1D individuals, eight out of the nine control participants of the original study, matched for age and BMI, also free of drugs with effect on sleep, no night- or shift-workers, and without previous diagnosis of sleep disorders, were included. They were submitted to a blood glucose test after fasting of 8 hours at a reference laboratory, as a requirement for inclusion in the control group.

### Instruments

Temperature data were collected every minute, during 10 consecutive days, and stored in the internal memory of the Tempatilumi (triple wrist equipment, with accelerometer, luxmeter and thermometer, produced by CEBrasil). All volunteers wore it continuously on the non-dominant wrist, removing it only for showers or any other water activity. An event button, pressed by the participants immediately before removing and when wearing it back, was available to identify unworn periods.

### Analysis

M10 and L5 were calculated^14^^-^^16^. The first (M10) corresponds to the mean of the 10 hours of wrist temperature values that surround the highest value, from all individuals and all days, while the second (L5) corresponds to the mean of the 5 hours temperature values around the lowest temperature value. The amplitude of the temperature oscillation was calculated as its coefficient of variation (relation between standard deviation and the mean) of the mean daily wrist temperature. All variables presented normal distribution (p-value greater than 0.05, Shapiro-Wilk’s W test) and, thus, differences between groups were analyzed using the Student T test for independent samples (*p*-value < 0.05 was considered significant). Variables are expressed as mean ± standard deviation.

### Ethics

Approval was obtained from the Ethics Committee for Research in Human Beings of the Instituto de Ciências Biomédicas, Universidade de São Paulo (number: 873/CEP), in addition to the approval from the partner institutions ADJ Diabetes Brasil, HC-FMUSP and InCor-HC-FMUSP. Informed consent was obtained from all participants.

## RESULTS

**Figure 1:**
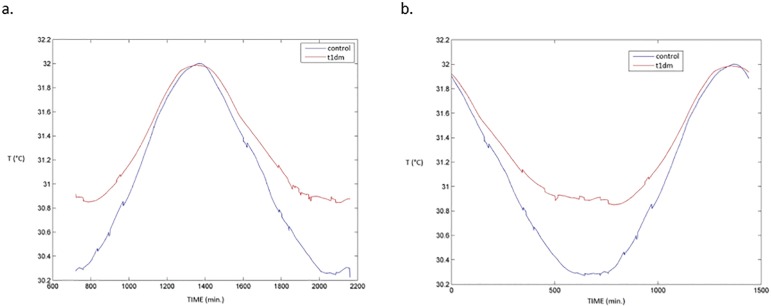
a. Graphic representation of the mean M10 for 10 days of collected wrist temperature; b. Graphic representation of the mean L5 for 10 days of collected wrist temperature (note that wrist temperature oscillates in opposition with the core body temperature6).

The T1D individuals’ mean age was 25.4±3.9 years, 5 were men and 7 women, their disease duration was of 11.3±6.7 years, and glycated hemoglobin (A1C) of 7.8±1.9%. The eight control individuals were 4 men and 4 women, 29.5±5.5 years old and presented a mean fasting glycaemia of 86±9 mg/dL. Although the mean wrist temperature and the M10 were not different when comparing data from both groups, the amplitude and L5 were clearly different, as seen on Table 1 and Figure 1. These results point toward a lower wrist temperature variability in T1D individuals, where the minimum temperature is higher than in control subjects, leading to lower temperature amplitude.

**Table 1:** Temperature analyses.

Temperature	T1D	Control	*p-*value
Mean	31.31±0.53°C	31.04±0.52°C	0.133
M10	31.96±0.51°C	31.94±0.62°C	0.465
L5	30.79±0.58°C	30.29±0.57°C	0.038
Amplitude	0.0142±0.0057°C	0.0203±0.0076°C	0.039

## DISCUSSION

Differently from the obtained results, lower M10 values would be expected to explain the lower wrist temperature amplitude in T1D individuals, since, according to previous findings, skin blood flow may be reduced in these individuals^3^. This has not appeared probably due to the fact that none of the participants presented chronic complications, such as peripheral or central neuropathy, and their glycemic control (A1C) was not severe. On the other hand, the higher L5 observed in the T1D group indicates a less efficient dissipation of heat in this population. This hypothesis is supported by different findings concerning reduced cutaneous vasodilatation and sweating in T1D individuals, which may limit their ability to adapt to different, especially extreme, external temperatures^3^.

This lower temperature amplitude, observed in T1D individuals, is similar to what was identified in the elderly^12^^,^^17^. We suggest that the coincidence in these different groups has in common the high oxidative stress process to which both are subject, possibly affecting the following structures: the circadian timing system (including especially the suprachiasmatic nuclei), the endogenous thermostat (hypothalamus), and the peripheral vascular and nervous systems. This higher oxidative stress alone and the results of processes that it triggers have been pointed out as the main cause of early chronic complications in individual with diabetes^18^^,^^19^. The temperature rhythm, with emphasis to its amplitude, acts as a signal, modulating other rhythms, including the sleep/wake cycle^11^^,^^12^. According to Van Someren^12^, increased skin temperature (heat loss, accompanied by the decrease of the core temperature) is associated with preparedness for sleep in brain structures such as the midbrain reticular formation, the hypothalamus and the cortex. Thus, thermoregulation alterations and its rhythm flatness, often seen is aging^11^^,^^12^, lead to shallow sleep and may help to explain sleep/wake and other rhythms impairments observed in individuals with diabetes^2^^,^^13^^,^^20^.

To our knowledge this is the first study on the rhythm of wrist temperature of T1D individuals. We suggest that future researches explore the core temperature rhythm of T1D individuals in parallel to the rhythm of wrist temperature, aiming to determine if this lower amplitude observed has a central determinant or if it is just the consequence of the skin blood flow reduction.
